# Whipple-Trias mit erhöhten und erniedrigten Insulinspiegeln

**DOI:** 10.1007/s00108-023-01473-6

**Published:** 2023-01-26

**Authors:** Thomas Karrasch, Bastian Eul, Stefan Gattenlöhner, Dagmar Steiner, Fritz Roller, Winfried Padberg, Andreas Schäffler

**Affiliations:** 1 Klinik und Poliklinik für Innere Medizin III (Endokrinologie, Diabetologie, Stoffwechsel und Ernährungsmedizin), Justus-Liebig-Universität Gießen (JLU) und Universitätsklinikum Gießen und Marburg (UKGM), Standort Gießen, Klinikstraße 33, 35392 Gießen, Deutschland; 2 Klinik und Poliklinik für Innere Medizin IV, Justus-Liebig-Universität Gießen (JLU) und Universitätsklinikum Gießen und Marburg (UKGM), Standort Gießen, Gießen, Deutschland; 3 Institut für Pathologie, Justus-Liebig-Universität Gießen (JLU) und Universitätsklinikum Gießen und Marburg (UKGM), Standort Gießen, Gießen, Deutschland; 4 Abteilung für Nuklearmedizin, Justus-Liebig-Universität Gießen (JLU) und Universitätsklinikum Gießen und Marburg (UKGM), Standort Gießen, Gießen, Deutschland; 5 Institut für diagnostische Radiologie, Justus-Liebig-Universität Gießen (JLU) und Universitätsklinikum Gießen und Marburg (UKGM), Standort Gießen, Gießen, Deutschland; 6 Klinik und Poliklinik für Allgemein‑, Viszeral‑, Thorax‑, Transplantations- und Kinderchirurgie, Justus-Liebig-Universität Gießen (JLU) und Universitätsklinikum Gießen und Marburg (UKGM), Standort Gießen, Gießen, Deutschland

**Keywords:** Insulinom, Hypoglykämie, Tumor, Merkel-Zell-Karzinom, IGF‑2, Insulinoma, Hypoglycemia, Tumor, Merkel cell carcinoma, IGF‑2

## Abstract

Eine 69-jährige Patientin und ein 70-jähriger Patient wurden mit rezidivierenden, schweren Hypoglykämien und klinischer Whipple-Trias aufgenommen. Bei der Patientin ließen erhöhte Spiegel an Insulin und C‑Peptid, ein pathologischer insulinogener Index und ein Fastentest an ein Insulinom denken, welches im ^68^Ga-DOTATOC-PET-CT im Pankreasschwanz detektiert wurde. Es bestand eine Koinzidenz mit einem neuroendokrinen Merkel-Zell-Karzinom. Bei dem Patienten zeigten sich hingegen supprimierte Spiegel an Insulin und C‑Peptid und es konnte eine tumorassoziierte, paraneoplastische Hypoglykämie infolge IGF-2-Sekretion gesichert werden, mit Nachweis eines erhöhten Glukoseverbrauchs in der Skelettmuskulatur (^18^F‑FDG-PET-CT).

## Abstract (en)

A 69-year-old female patient and a 70-year-old male patient were admitted to hospital with recurrent, severe hypoglycemic episodes and a typical manifestation of Whipple’s triad. In the female, elevated levels of insulin, C‑peptide and pro-insulin together with pathological findings during a fasting test proved the presence of an insulinoma, which could be detected by Ga-68-DOTATOC-PET-CT in the pancreas. There was a very rare co-existence of a neuroendocrine Merkel cell carcinoma. In the male, levels of insulin and C‑peptide were suppressed and a diagnosis of paraneoplastic hypoglycemia by IGF‑2 secretion was made with increased glucose disposal in skeletal muscle proven by ^18^F‑FDG-PET-CT.

## Anamnese und körperliche Untersuchung

### 69-jährige Patientin.

Die 69-jährige Patientin in gutem Allgemeinzustand und adipösem Ernährungszustand (BMI 37 kg/m^2^, RR 122/73, HF = 82/min) stellte sich zur Abklärung rezidivierender, schwerer, symptomatischer Hypoglykämien vor. Die neuroglykopenischen und autonomen Symptome traten zu jeder Tageszeit auf, bestanden seit 6 Monaten, äußerten sich durch Übelkeit, Tremor, Schwäche, Palpitationen, Schweißausbrüche und waren durch Glukosezufuhr stets zu unterbrechen. In den letzten 6 Monaten hatte die Patientin aus Angst vor weiteren Hypoglykämien vermehrt Kohlenhydrate zugeführt, auch nachts, was zu einer Gewichtszunahme von 8 kg führte. Zuletzt war eine Einweisung bei Somnolenz und einer Hypoglykämie von 25 mg/dl über den Notarzt erfolgt. Der Leidensdruck der Patientin war hoch und die psychologische Anamnese erbrachte keine Hinweise auf eine Selbstmedikation mit Insulinen oder Sulfonylharnstoffen. Medikation: L‑Thyroxin 100 μg (1-0-0), Amlodipin 5 mg (1-0-0). Vorerkrankungen: Z.n. Thyreoidektomie bei Struma multinodosa, Arterielle Hypertonie, Z. n. Merkel-Zell-Karzinom des linken Unterarms (kurative Resektion und Radiatio).

### 70-jähriger Patient.

Der 70-jährige Patient in gutem Allgemeinzustand und adipösem Ernährungszustand (BMI 31 kg/m^2^, RR 140/87, HF = 69/min) wurde vom Notarzt bei einer Serumglukose von 36 mg/dl sowie Somnolenz und Tremor eingewiesen. Die Symptomatik konnte durch Glukosezufuhr behoben werden. Im stationären Verlauf traten rezidivierend schwere symptomatische Hypoglykämien auf, die eine intravenöse Glukosezufuhr erforderlich machten. Es bestanden keine Hinweise auf die Einnahme von blutzuckersenkenden Medikamenten. An Vorerkrankungen waren bekannt: Z. n. Myokarditis mit Ausheilung. Medikation: keine.

### Vergleichende Wertung:

*Somit bestand bei beiden Patienten klinisch eine ausgeprägte Whipple-Trias* (= Symptome einer Hypoglykämie + gemessene Hypoglykämie + Besserung auf Glukosezufuhr).

## Laborchemische und endokrinologische Diagnostik

### 69-jährige Patientin

#### Blutzuckertagesprofile mit Point-of-care-Messungen unter stationärer Überwachung

Bei Aufnahme erfolgte ein Point-of-care-Blutzuckertagesprofil mit der Anordnung, im Falle von symptomatischen Hypoglykämien eine Messung von Glukose, Insulin und C‑Peptid im Zentrallabor durchzuführen und eine Serumprobe zu asservieren. Unter stationären Bedingungen traten mehrfache Hypoglykämien < 60 mg/dl auf, die sich symptomatisch durch Glukosezufuhr kontrollieren ließen.

#### Berechnung des *insulinogenen Index* und des *korrigierten Turner-Index*

Bei einer symptomatischen Hypoglykämie mit einer Serumglukose von 56 mg/dl (*60–100*) konnten simultan ein inadäquat hoher Insulinspiegel von 30,2 μU/ml (*3,0–25,0*) und ein inadäquat hohes C‑Peptid von 4,43 ng/ml (*0,9–4,0*) nachgewiesen werden. Der Proinsulinspiegel war mit 9,6 pmol/l (1,8–11) in der Hypoglykämie inadäquat erhöht (> 5 verdächtig auf endogenen Hyperinsulinismus). Somit war eine exogene Insulinapplikation ausgeschlossen. Der errechnete *insulinogene Index* betrug 0,54 (> 0,5 verdächtig auf endogenen Hyperinsulinismus), der *korrigierte Turner-Index* betrug 116 (> 100 verdächtig auf endogenen Hyperinsulinismus, auch bei Adipositas). Eine weitere Hypoglykämie von 58 mg/dl bei einem Insulin von 48,2 μU/ml und einem C‑Peptid von 4,98 ng/ml erbrachte ebenfalls einen pathologischen *insulinogenen Index* von 0,83 und einen *korrigierten Turner-Index* von 172.

#### Durchführung eines 24 h-Fastentests („Hungerversuch“)

Unter Durchführung eines 24 h-Fastentests konnte nach 22 h eine Hypoglykämie von 38 mg/dl bei inadäquat hohem Insulinspiegel (23,3 μU/ml) und inadäquat hohem C‑Peptid von 3,95 ng/ml dokumentiert werden. Der *insulinogene Index* betrug hier 0,61 und der *korrigierte Turner-Index* 291, beide also hoch pathologisch. Somit bestand der hochgradige Verdacht auf ein Insulinom. Der neuroendokrine Tumormarker NSE war mit 32,7 ng/ml erhöht (0–18,3).

### 70-jähriger Patient

In Phasen der Hypoglykämie erfolgten wiederholte Bestimmungen von Glukose (z. B. 36 mg/dl), Insulin (z. B. 0,2 μU/ml) und C‑Peptid (z. B. 0,42 ng/ml). Bei supprimierten Insulin- und C‑Peptid-Werten waren demnach ausgeschlossen: Applikation von Insulin oder Insulin-Sekretagoga, endogener Hyperinsulinismus, Insulinom. Zudem konnten eine Nebennierenrindeninsuffizienz, eine Hypophyseninsuffizienz, ein Alkoholabusus, eine Leberzirrhose und eine Niereninsuffizienz ausgeschlossen werden.

Um die Differenzialdiagnose einer tumorinduzierten Hypoglykämie im Sinne einer „*n*on-*i*slet *c*ell *t*umor *h*ypoglycemia“ (NICTH) voranzutreiben, erfolgte eine Bestimmung des IGF‑1 mit 150 ng/ml (24–200 ng/ml), des IGFBP‑3 mit 4,17 mg/l (1,9–5,5 mg/l) und des IGF‑2 mit 494 ng/ml (429–755 ng/ml), während für das sogenannte „big IGF-2“ aktuell keinerlei Assays zur Verfügung standen. Das IGF-2/IGF-1-Verhältnis lag bei 3,3 (> 3 bei IGF-2-induzierter, tumorassoziierter Hypoglykämie).

#### Vergleichende Wertung:

*Somit bestand bei der 69-jährigen Patientin ein endogener Hyperinsulinismus mit erhöhten Werten für Insulin und C‑Peptid und daher der Verdacht auf ein Insulinom. Bei dem 70-jährigen Patienten hingegen lagen supprimierte Insulin- und C‑Peptid-Werte vor mit V.* *a. tumorassoziierte Hypoglykämien im Sinne einer „non-islet cell tumor hypoglycemia“ (NICTH), am ehesten aber IGF-2-induziert im Sinne eines paraneoplastischen Syndroms.*

## Bildgebende Diagnostik

### 69-jährige Patientin

#### MRT des Abdomens

**Figure 1:**
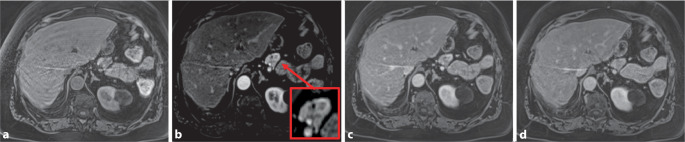

In der MRT des Abdomens (Abb. 1) zeigte sich eine 15 mm große, zirkulär hypervaskularisierte Läsion im Pankreasschwanz. Die Raumforderung war im Vergleich zum Pankreasparenchym leicht hypointens in der Nativsequenz und leicht hyperintens in der portal-venösen Phase.

#### PET-CT mittels ^68^Ga-DOTATOC

**Figure 2:**
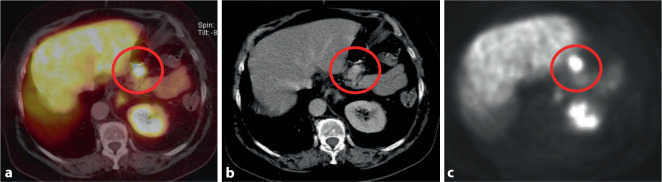

Die CT zeigte eine 15 mm große, hypervaskularisierte Raumforderung des Pankreasschwanzes (Abb. 2). In der PET-CT fand sich eine deutliche fokale Anreicherung des Nuklids in der Raumforderung, beweisend für einen somatostatinrezeptorexprimierenden Tumor (Insulinom; Abb. 2).

### 70-jähriger Patient

#### PET-CT

**Figure 3:**
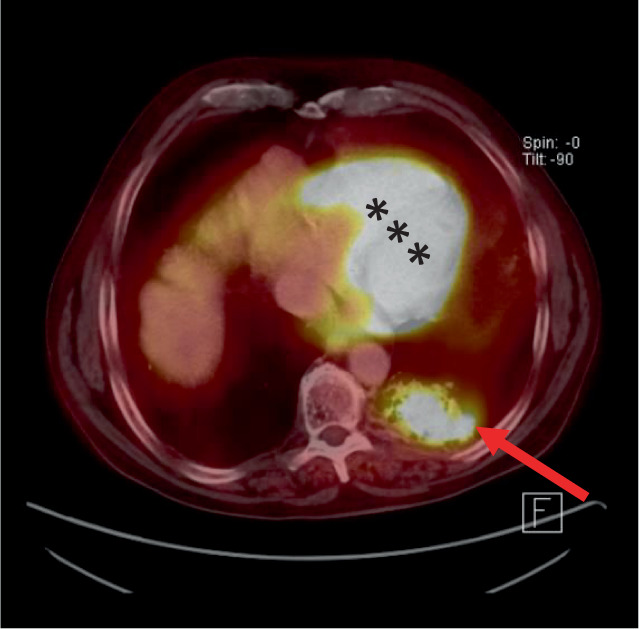

Im PET-CT (Abb. 3) konnte eine tumorsuspekte Läsion des linken Unterlappensegments der Lunge nachgewiesen werden.

## Diagnosen

69-jährige Patientin:

Insulinom des Pankreasschwanzes mit endogenem Hyperinsulinismus

70-jähriger Patient:

Tumorassoziierte Hypoglykämien bei malignem Tumor der Lunge

## Therapie und Verlauf

### 69-jährige Patientin.

**Figure 4:**
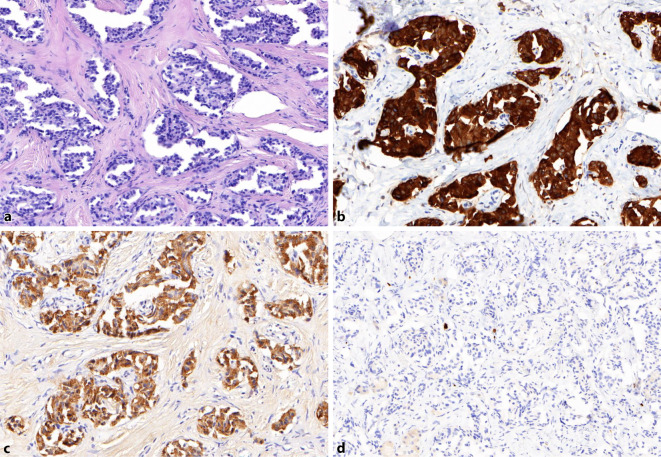

Es wurde die Indikation zur Resektion des Pankreastumors bei V. a. Insulinom gestellt. Überbrückend wurde während der Diagnostik und bis zur Operation eine erfolgreiche Kontrolle der Hypoglykämien durch Gabe von Diazoxid (3 × 50 bis 3 × 100 mg tgl. oral) erreicht. Die histologische Aufarbeitung (Abb. 4) ergab ein Insulinom (pT1, Ø 1,8 cm, Nx, L0, V0, G1, MIB‑1 von 1 %) mit starker Positivität für Insulin und Synaptophysin (sowie für Chromogranin A, CD56, INSM1). Postoperativ traten keine Hypoglykämien mehr auf. Klinisch und laborchemisch bestanden keinerlei Anhaltspunkte für ein MEN-1-Syndrom (endokriner Pankreastumor, primärer Hyperparathyreoidismus, Hypophysentumor).

### 70-jähriger Patient.

**Figure 5:**
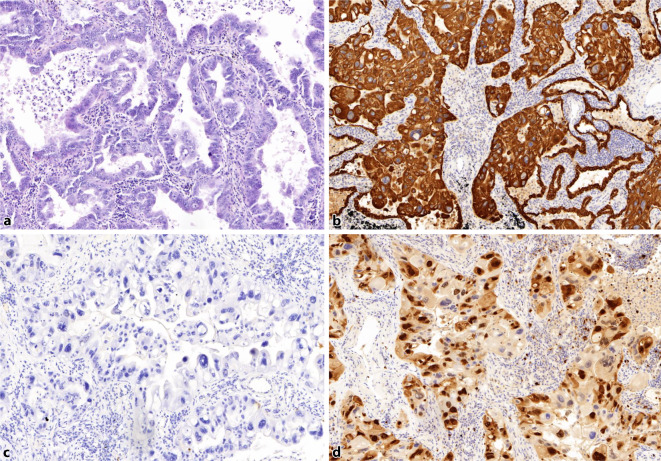

In einer initialen Stanzbiopsie der Lungenraumforderung zeigte sich ein schlecht differenziertes nichtkleinzelliges Karzinom. Die endgültige Histologie/Immunhistochemie nach Resektion (Abb. 5) erbrachte ein 4,1 cm großes, schlecht differenziertes, stark proliferierendes bronchopulmonales Adenokarzinom (NSCLC) mit Riesenzellkomponente (MIB-1-Index 40 %) im Tumorstadium pT3 (m), pN1 (1/10), L0, V0, G3/4 bei Nachweis von Positivität für Cytokeratin‑7 und Negativität für „thyroid transcription factor-1“ (TTF1).

**Figure 6:**
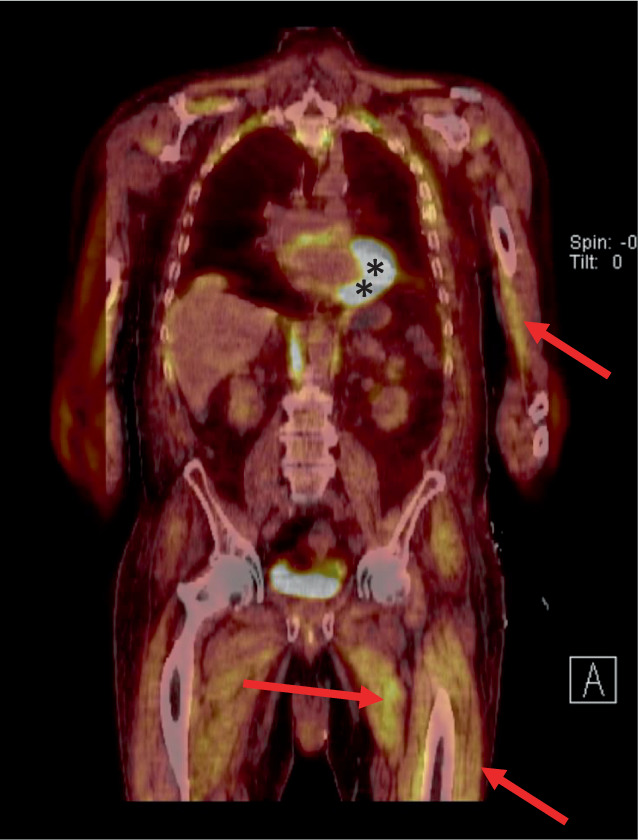

Nach Resektion des Tumors bestanden die symptomatischen Hypoglykämien zunächst weiter, sodass ein massenhafter Glukoseverbrauch durch die Tumorlast selbst ausgeschlossen werden konnte und somit der Verdacht auf eine IGF-2-induzierte Hypoglykämie im Sinne eines paraneoplastischen Syndroms bestand. Da in diesem Falle ein peripher erhöhter Glukoseverbrauch der Muskulatur in Ruhe zu finden sein müsste, erfolgte postoperativ eine ^18^F‑FDG-PET-CT (Fluordesoxyglukose). Diese konnte eine pathologisch gesteigerte Aufnahme der markierten Glukose in die quergestreifte Muskulatur nachweisen (Abb. 6). Es wurde eine Chemotherapie mit Carboplatin, Pemetrexed und Pembrolizumab eingeleitet, worunter die Hypoglykämien sistierten.

## Diskussion

Bei der 69-jährigen Patientin mit Insulinom bestand anamnestisch der Zustand nach Operation und Radiatio eines sehr seltenen Merkel-Zell-Karzinoms (MCC) (Synonym: kutanes, neuroendokrines Karzinom; Ursprung: neuroendokrine Zellen der Haut). Dies ist eine extrem seltene Koinzidenz, welche möglicherweise kausal-pathogenetische Implikationen aufweist. Ein „case report“ aus dem Jahr 2005 konnte erstmals die Koinzidenz eines Insulinoms (Inzidenz: 4/1.000.000 Personen/Jahr) mit einer pankreatischen Metastase eines MCC (Inzidenz: 7/1.000.000 Personen/Jahr) belegen, was die Beschreiber entweder als zufällige Koinzidenz oder als bislang nicht bekanntes neuroendokrines Tumorsyndrom werteten [1]. Im hier vorgestellten Fall war eine pankreatische Metastase des MCC ausgeschlossen. Für die Diagnose eines MCC werden immunhistochemische, neuroendokrine Marker wie Chromogranin A, Synaptophysin, CD56 oder neuronenspezifische Enolase (NSE) benötigt. „Insulinoma-associated-1“ (INSM1) konnte in den letzten Jahren als zusätzlicher immunhistochemischer Marker des MCC etabliert werden [2, 3]. INSM1 ist ein neuroendokriner Transkriptionsfaktor mit einer Zinkfinger-DNA-bindenden Domäne, welcher initial aus „insulinoma libraries“ isoliert wurde. INSM1 bindet an Cyclin D1 und reguliert den Zellzyklus, die neuroendokrine zelluläre Differenzierung und Proliferation [4, 5]. Aktuelle Daten belegen, dass INSM1 den konventionellen neuroendokrinen Markern überlegen erscheint und in der Differenzierung pankreatischer NET von Non-NET INSM1 eine 100 %ige Sensitivität und Spezifität [6] aufweist. Somit ist es nicht ganz abwegig, dass die INSM1-Expression bei einem MCC mit der synchronen oder metachronen Entwicklung eines Insulinoms kausal-pathogenetisch verknüpft sein könnte oder dass beide Entitäten eine bislang unbekannte gemeinsame genetische Basis im Sinne eines neuroendokrinen Tumorsyndroms haben könnten.

Die Ursachen für Hypoglykämien sind extrem vielfältig, ebenso die klinischen Beschwerden, welche mit einer Hypoglykämie vereinbar wären. Daher ist die sorgfältige Dokumentation einer Whipple-Trias bedeutsam und ein erster Schritt vor den diagnostischen Maßnahmen. Die *Clinical Practice Guideline* der *Endocrine Society* gibt einen sehr guten Überblick über Evaluation und Management von Hypoglykämien [7]. Neben Patientenselbstmessungen oder dezentralen Point-of-care-Glukosemessungen ist es mandatorisch, die Hypoglykämien im Zentrallabor messtechnisch sicher zu erfassen. In der Präanalytik ist darauf zu achten, ein Natriumfluorid(NaF)-Röhrchen zu verwenden (Glykolysehemmer). Die simultane Bestimmung des Insulins stellt die Weichen dahingehend, ob eine insulininduzierte Hypoglykämie vorliegt. Hier unterscheidet man zwischen endogenem Hyperinsulinismus (z. B. Insulinom, Nesidioblastose, reaktive postprandiale Hyperglykämie), Einnahme insulinotrop wirkender Substanzen (Sulfonylharnstoffe, Glinide) oder exogen zugeführtem Insulin. Die simultane Bestimmung des C‑Peptids (und des Proinsulins) ist wichtig, da nur so zwischen endogen induzierter Insulinsynthese (Insulin, C‑Peptid, Proinsulin erhöht) und exogen zugeführtem Insulin (Insulin erhöht, C‑Peptid/Proinsulin supprimiert) unterschieden werden kann. Sind die Insulin‑, C‑Peptid- und Proinsulinspiegel bei einer Hypoglykämie supprimiert, so kommen vielfältige andere Ursachen infrage (Leberzirrhose, Niereninsuffizienz, nichtinsulinotrope blutzuckersenkende Substanzen, Nebennierenrinden- und Hypophyseninsuffizienz, Kachexie, Kurzdarmsyndrom, chronisch-entzündliche Darmerkrankungen, Alkoholismus, Sepsis, Malaria etc.). Auch tumorassoziierte Hypoglykämiesyndrome („*n*on-*i*slet *c*ell *t*umor *h*ypoglycemia“ [NICTH]) können ursächlich sein [8, 9], entweder im Sinne eines gesteigerten Glukoseverbrauchs des Tumors selbst bei sehr großen, schnell proliferierenden Tumoren (z. B. Sarkome) oder als paraneoplastisches Syndrom durch Sekretion blutzuckersenkender Faktoren wie IGF‑2 mit erhöhtem Glukoseverbrauch in der Muskulatur [10]. IGF-2-assoziierte Hypoglykämien sind bei einer Vielzahl unterschiedlicher Tumoren beschrieben, auch der Lunge, insgesamt jedoch sehr selten.

## Fazit für die Praxis

Kaum ein anderes Symptom ist so häufig, so interdisziplinär und hat derartig multifaktorielle Ursachen wie die Hypoglykämie. Bei Hypoglykämien im Rahmen einer Whipple-Trias ist neben der sorgfältigen Anamnese und Medikamentenerfassung die simultane Bestimmung von Serumglukose, Insulin und C‑Peptid initial weichenstellend, in der Präanalytik gibt es viele kritische Punkte zu beachten. Die Berechnung des insulinogenen Index/korrigierten Turner-Index ist wegweisend für die Verdachtsdiagnose/Differenzialdiagnose eines endogenen Hyperinsulinismus (z. B. Insulinom). Die Abklärung von tumorassoziierten Hypoglykämien kann sehr aufwendig und schwierig verlaufen.
